# Potential Muscle-Related Biomarkers in Predicting Curve Progression to the Surgical Threshold in Adolescent Idiopathic Scoliosis—A Pilot Proteomic Study Comparing Four Non-Progressive vs. Four Progressive Patients vs. A Control Cohort

**DOI:** 10.3390/jcm10214927

**Published:** 2021-10-25

**Authors:** Yujia Wang, Huanxiong Chen, Jiajun Zhang, Tsz-ping Lam, A.L.H. Hung, J.C.Y. Cheng, W.Y.W. Lee

**Affiliations:** 1 Department of Orthopaedics and Traumatology, SH Ho Scoliosis Research Laboratory, Joint Scoliosis Research Center of the Chinese University of Hong Kong and Nanjing University, The Chinese University of Hong Kong, Hong Kong 999077, China; wangyujia716@foxmail.com (Y.W.); chenhuanxiong@hainmc.edu.cn (H.C.); zhangjiajun1@genomics.cn (J.Z.); tplam@cuhk.edu.hk (T.-p.L.); lhhung@ort.cuhk.edu.hk (A.H.); 2 Li Ka Shing Institute of Health Sciences, The Chinese University of Hong Kong, Hong Kong 999077, China; 3 Department of Spine and Osteopathic Surgery, The First Affiliated Hospital of Hainan Medical University, Haikou 570102, China

**Keywords:** scoliosis, iTRAQ, α-actin, progressive, differentially expressed proteins

## Abstract

Previous studies have reported abnormal muscle morphology and functions in patients with adolescent idiopathic scoliosis (AIS). To answer whether such abnormalities could be reflected in their circulation and their clinical implication for predicting curve progression to the surgical threshold, this preliminary study explored the presence of baseline muscle-related proteins and their association with curve progression. Plasma samples were collected at the first clinical visit for AIS, with patients divided into non-progressive or progressive groups (*N* = four and four) according to their Cobb angle in six-year follow-ups, with age- and sex-matched healthy subjects (*N* = 50). Then, the samples were subjected to isobaric tags for relative and absolute quantitation (iTRAQ) for global comparison of untargeted protein expression. Seventy-one differentially expressed proteins (DEPs) were found elevated in progressive AIS. Functional analysis showed that 18 of these are expressed in muscles and play an essential role in muscle activities. Among the muscle-related DEPs, α-actin had the highest fold change in progressive/non-progressive groups. This preliminary study firstly suggested higher circulating levels of muscle structural proteins in progressive AIS, indicating the likelihood of structural damage at the microscopic level and its association with progression to the surgical threshold. Further studies with larger sample sizes are warranted to validate these novel candidates for early diagnosis and predicting progression.

## 1. Introduction

Adolescent idiopathic scoliosis (AIS) is the most common type of three-dimensional structural deformity occurring during the puberty growth period, with a prevalence of 1~4% worldwide. AIS is more prevalent in girls than in boys [1]. For example, a five-year idiopathic scoliosis screening program of 255,875 children aged 11~14 years old in Japan reported that the prevalent ratio of girls to boys was 11:1 [2]. Our local scoliosis study on 115,190 fifth grade children indicated that the prevalence ratio of girls to boys with a Cobb angle ≥10° was 2.7 by the age of 19 years, and the ratio increased to 4.5 and 8.1 with a Cobb angle ≥20° and ≥40°, respectively [3]. Unlike congenital, neuromuscular, and other types of scoliosis, the etiopathogenesis of AIS is largely unknown [1]. Bracing is an evidence-based effective treatment for patients with a Cobb angle ≥20° by means of preventing curve progression [4]. However, wearing braces could cause a negative cosmetic appearance, poor self-esteem, and functional discomfort [5], resulting in insufficient wearing time and thus affecting the effectiveness of this treatment. Those that develop a Cobb angle of major curve >40~45°in the thoracolumbar region, or >50° in the thoracic region, might accept invasive surgery to correct the anatomical deformation and to reduce the risk of further progression during adulthood. However, the potential complications of surgery, including partial or complete loss of neurological function, infection, implant failure or pseudoarthrosis, recurrence, or additional deformity, should be carefully taken into consideration [1]. Current understanding of the etiopathogenesis is limited. Investigation on unexplored areas is worthwhile to develop more effective prognostication and treatment.

AIS patients have been reported to have weaker muscle strength than healthy subjects of a similar age and sex [6,7]. Decreased respiratory muscle strength in patients with AIS has been described in pulmonary function studies [6,8]. The posterior paraspinal muscles, including the multifidus and erector spinal muscle, provide dynamic stability to the spinal column [9], and its imbalance has been postulated to contribute to the initiation and/or progression of spinal deformity in AIS [10,11]. Previous studies have reported abnormal and asymmetric muscle phenotypes in concave and convex side paraspinal muscles of AIS patients, including electromyography (EMG) activities, muscle volume, muscle fiber types, and fatty and fibrosis infiltration [12,13,14,15,16]. Additionally, one recent study demonstrated a significantly lower density of activated satellite cells for fiber type I in AIS patients when compared to non-scoliosis controls, and the curve severity appeared to be associated with the density of satellite cells and other histological parameters such as cross-sectional areas of muscle fiber and myonuclear density [17]. Collectively, these findings suggest that patients with AIS have generalized muscle dysfunction, which is potentially associated with the curve severity. Until now, whether muscle abnormalities could be a predictive value for AIS onset or curve progression remained unexplored.

In AIS, the current studies on paraspinal muscles rely heavily on muscle biopsies, which is a major research hurdle due to ethical concerns and the scarcity of muscle biopsies from healthy control subjects or AIS patients with mild curvature for fair comparison. Thus, less invasive tests are widely used to reveal muscle-related changes at earlier time points and for longitudinal study. Blood samples are a surrogate for systemic phenotype research, which allows for biomarker discovery. Currently, the advancement in “omics” research enables researchers to quantify a large amount of proteins/peptides and metabolites in the circulation in a non-targeted and unbiased manner, resulting in identification of numerous AIS-related predictive and prognostic biomarkers [18]. A recent metabolomics study that performed UPLC/QTOF-MS (ultra-high-performance liquid chromatography coupled with quadrupole time-of-flight mass spectrometry) analysis revealed differential serum lipid metabolism profiles in patients with AIS [19]. This study suggested disrupted glycerophospholipid, glycerolipid, and fatty acid metabolism in AIS patients when compared to age-matched healthy controls, and provided a list of metabolites for diagnostic biomarkers. Another two proteomic studies described the differential circulating proteomes in AIS. Shen et al. [20] compared the plasma samples derived from four AIS patients and four healthy controls using 10-plex tandem mass tag (TMT)-based quantitative MS analysis. They identified several proteins correlating with the differential gut microbiota species in AIS patients. Makino et al. [21] employed two-dimensional fluorescence difference gel electrophoresis (2D-DIGE) quantitation followed by an MS-based proteins identification strategy to compare pooled plasma samples from five severe AIS and five non-AIS control subjects, revealing the association between vitamin D binding protein and coagulation-related proteins with AIS pathogenesis. However, these studies did not step further to explore the link between the acquired proteomic data and the risk of curve progression in AIS, specifically exploring biomarkers with respect to muscles. Therefore, the current study aimed to compare proteomic profiles among healthy girls and AIS subjects with either remaining curvature or progressing into severe cases, further providing a candidate list of muscle-related DEPs for functional and clinical validation.

In this study, we aimed to (1) conduct a quantitative proteomic study screening the plasma samples from healthy controls and AIS patients, who were further divided into non-progressive and progressive subgroups regarding their curve progression in a longitudinal follow-up; (2) compare the proteomic profiles among these three groups and filter out a list of candidate muscle-related proteins.

## 2. Materials and Methods

### 2.1. Subjects Recruitment and Blood Taking

As referred by the local population-based School Scoliosis Screening Service, students of the fifth grade or above with a maximal Cobb angle of ≥20° were referred to our scoliosis special clinic [3]. As the prevalence of AIS is likely linked to sex and there is a higher risk of curve progression in girls, female subjects with AIS with a maximal Cobb angle of <30°at their first clinical visit were recruited from our scoliosis clinic. Patients with other types of scoliosis with known causes or with congenital deformities, neuromuscular diseases, autoimmune disorders, endocrine disturbances, or medical conditions that affect the bone metabolism were excluded, as previously reported [22]. Non-progressive subjects prescribed with bracing or any other treatment that might interfere with curve progression during the follow-up were excluded.

**Figure 1:**
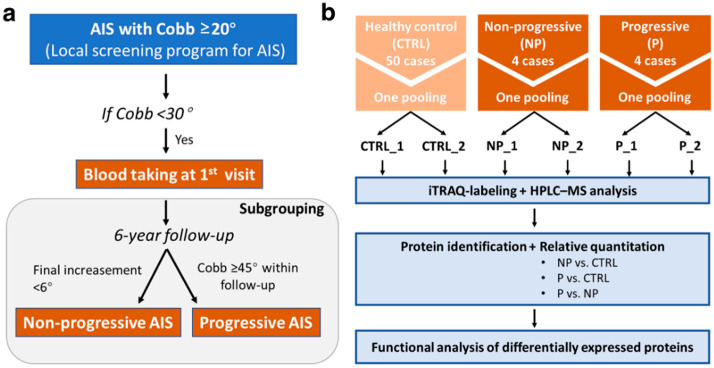
Proteomic analysis of the plasma in the healthy control (CTRL) vs. non-progressive (NP) vs. progressive (P) AIS groups. (**a**) Illustration of the selection criteria for the AIS groups. (**b**) Main procedures of sample pooling, iTRAQ labeling, HPLC–MS analysis, and bioinformatic analysis.

Blood samples and clinical data from the CAL cohort (NCT01103115) were used. The Cobb angle of a major curve was measured via standard standing posterior–anterior radiography of the whole spine at the first visit and at six-month intervals for six years (reaching skeletal maturity). All of the subjects were regularly followed, observed, and/or treated with bracing or surgical correction according to standard clinical practice (CREC Reference Number: 2009.491-T). Four subjects with curve progression that increased less than 6° in the follow-up period were defined as the non-progressive (NP) group [23], and four subjects that had reached the surgical threshold (≥45°) at any time point within the follow-up period were defined as the progressive (P) group. The classification criteria for the NP and P groups are illustrated in Figure 1a. Basic anthropometric data were measured with standardized methodology. Tanner staging and age at menarche were recorded. The body composition parameters were determined via bioelectrical impedance analysis (BIA, InBody 720, Biospace, Seoul, Korea). Handgrip strength was assessed with portable dynamometers (Nakamura Scientific Co., Ltd., Tokyo, Japan) three times using the dominant and non-dominant hands, and the mean of the three measurements was calculated.

A total of 50 age-matched healthy Chinese girls were recruited randomly from local secondary schools to serve as the control (CTRL) group. Clinical examination was carried out by experienced orthopedic surgeons to exclude spinal deformities. The basic information for the CTRL, AIS NP, and AIS P groups is described in Table 1.

Peripheral venous blood samples (2 mL) were collected from the participants’ arms at their first visit to our scoliosis special clinic. The blood was centrifuged at 4 °C, 3000× *g* for 10 min. The plasma were aliquoted to minimize the freeze–thaw cycle and stored at −80 °C for further analysis.

### 2.2. Sample Preparation for iTRAQ-Based Proteomic Analysis

Isobaric tags for relative and absolute quantitation (iTRAQ)-based [24] proteomic analysis allows relative quantitation, comparing the proteomic profiles of plasma from three groups. Considering the limitation of the maximal eight samples for one 8-plex analysis and the sample sizes of the designed comparison groups (CTRL: *N* = 50; NP: *N* = 4; P: *N* = 4), the strategy of groupwise pooling followed by technical duplicates was adopted (Figure 1b). An equal volume (300 μL) of each plasma sample within each group was pooled into one mixture and further divided into two duplicates, resulting in six plasma samples (CTRL_1, CTRL_2, NP_1, NP_2, P_1, and P_2). The duplicates in this study allowed for a decrease in the variance due to the technical error of the experimental technique.

High abundance protein depletion was carried out with a ProteoMiner Protein Enrichment Kit (Cat. # 163-3007; Bio-Rad laboratories, Hercules, CA, USA) according to the manufacturer’s instructions. The proteins were dissolved in lysis buffer consisting of 8 M urea for denaturation afterwards. For cysteine alkylation, protein lysis was further subjected to 10 mM dithiothreitol (DTT, Cat. #10197777001, Sigma-Aldrich, Burlington, MA, USA) with incubation at 57 °C for 45 min, followed by 10 mM iodoacetamide (IAM, Cat. #I1149-5G, Sigma-Aldrich, Burlington, MA, USA) and incubation at room temperature in the dark for 1 h. A Bradford assay was used to determine the total protein concentration, for which 50 μg of protein of each sample was diluted four times with 100 mM triethylammonium bicarbonate (TEAB, Cat. #90114, Thermo Fisher Scientific, Waltham, MA, USA). Then, the protein solution was subjected to protein digestion with Trypsin Gold (Cat. #V5117; Promega, Madison, WI, USA). Trypsin Gold was added at a 1:40 (*w*/*w*) enzyme/protein ratio for further incubation at 37 °C for 18 h. HCl was added to a final concentration of 50 mM to stop the tryptic digestion. After trypsin digestion, the peptides were desalted with a Strata X C18 column (Phenomenex, Torrance, CA, USA) according to the manufacturer’s protocol, and then vacuum-dried.

The digested peptides from 50 μg of protein were dissolved in 30 μL of 0.5 M TEAB and subjected to peptide labeling with an iTRAQ Reagent 8-plex Kit (SCIEX, Framingham, MA, USA) according to the manufacturer’s instructions [25]: 113 and 114 for the CTRL group, 116 and 117 for the NP group, and 119 and 121 for the P group. The labeled peptides were combined and desalted with a Strata X C18 column followed by vacuum-drying. The peptides were subjected to a Shimadzu LC-20AB high-performance liquid chromatography (HPLC) pump system (Shimadzu, Kyoto, Japan) coupled with a high pH reversed-phase (RP) column to separate into 20 fractions. The following gradient was used for fractionation. The peptides were reconstituted with buffer A (5% acetonitrile (Cat. #900667-4 × 4L, Sigma-Aldrich, Burlington, MA, USA), 95% H_2_O, pH 9.8) to 2 mL and loaded onto a column containing 5 μm of particles (Phenomenex, Torrance, CA, USA). The peptides were separated at a flow rate of 1 mL/min with a gradient of 5% buffer B (5% H_2_O, 95% acetonitrile, pH 9.8) for 10 min, 5~35% buffer B for 40 min, and 35~95% buffer B for 1 min. The system was then maintained in 95% buffer B for 3 min and decreased to 5% within 1 min, before equilibrating with 5% buffer B for 10 min. Elution was monitored by measuring the absorbance at 214 nm, and fractions were collected every 1 min. The eluted peptides were pooled as 20 fractions and vacuum-dried. Each fraction was resuspended in 2% acetonitrile and 0.1% formic acid (Cat. #33015-500ML, Sigma-Aldrich, Burlington, MA, USA), and centrifuged at 20,000× *g* for 10 min. The supernatant was subjected to the following HPLC–MS analysis.

### 2.3. HPLC–MS and Bioinformatic Analysis

Each fraction was loaded onto a C18 trap column using a LC-20AD nano-HPLC instrument (Shimadzu, Kyoto, Japan) by an autosampler. Then, the peptides were eluted from the trap column and separated by an analytical C18 column (inner diameter of 75 μm) packed in-house, and then subjected to MS analysis with a TripleTOF 5600 System (SCIEX, Framingham, MA, USA) equipped with a Nanospray III source (SCIEX, Framingham, MA, USA). The following gradient was used for elution of the peptides for MS analysis: buffers A and B for elution were 2% acetonitrile with 0.1% formic acid, and 98% acetonitrile with 0.1% formic acid, respectively. The gradient was run at 300 nL/min, starting from 8~35% of buffer B for 35 min, then up to 60% for 5 min, maintained at 80% B for 5 min, and finally returned to 5% for 0.1 min and equilibrated for 10 min.

For the MS setting, the high-sensitivity mode was used for the whole data acquisition. The accumulation time for MS1 was 250 ms, and the mass ranges were from 350 to 1500 Da. Based on the intensity in the MS1 survey, as many as 30 product ion scans were collected if exceeding a threshold of 120 counts/s and with charge-state 2 + to 5 +, dynamic exclusion was set for 1/2 of the peak width (12 s). For iTRAQ data acquisition, the collision energy was adjusted to all precursor ions for collision-induced dissociation, and the Q2 transmission window for 100 Da was 100%. The raw MS/MS data were converted into MGF format by the ProteoWizard tool msConvert, and the exported files were searched using Mascot version 2.3.02 against the selected database Swissprot (Homo_sapiens, 201704). The fragment and peptide mass tolerance were set at 0.1 and 0.05 Da, respectively. The variable modifications were Oxidation (M) and iTRAQ8plex (Y), while the fixed modifications were Carbamidomethyl (C), iTRAQ8plex (N-term), and iTRAQ8plex (K). One missed cleavage was allowed. The false discovery rate (FDR) at 1%, which was based on the picked protein FDR strategy [26], was used for cut-off. Automated software IQuant Protein Quantification [27] was used to analyze the labeled peptides with isobaric tags. In this project, we set NP_1/CTRL_1, NP_2/CTRL_1, NP_1/CTRL_2, NP_2/CTRL_2, P_1/CTRL_1, P_1/CTRL_2, P_2/CTRL_1, P_2/CTRL_2, P_1/NP_1, P_1/NP_2, P_2/NP_1, and P_2/NP_2 as the comparison groups. The mean ratio of the protein expression in the pairwise comparison groups was calculated. For example, the mean ratio of NP vs. CTRL = (NP_1/CTRL_1 + NP_1/CTRL_2 + NP_2/CTRL_1 + NP_2/CTRL_2)/4. A protein with a mean ratio ≤0.83 or ≥1.2, a *p*-value < 0.05 by an independent *t*-test was considered as differentially expressed protein (DEP).

A Venn diagram for overlapping analysis was performed using Venny v2.1 [28]. The functions of the DEPs were analyzed using Protein Analysis Through Evolutionary Relationships (PANTHER) v15.0 [29] to demonstrate their Gene Ontology (GO) profiles. Detailed GO term descriptions at the level of DIRECT were obtained using the Database for Annotation, Visualization, and Integrated Discovery (DAVID) v6.8 [30]. A protein–protein interaction (PPI) network of the DEPs was constructed using the online Search Tool for the Retrieval of Interacting Genes (STRING) database [31]. The minimum required interaction score was set to medium confidence (0.4). The PPI results were exported and subjected to Cytoscape v3.7.2 for visualization. The other results were visualized using PRISM v7.03. The mass spectrometry proteomics data have been deposited to the ProteomeXchange Consortium via the PRIDE [32] partner repository with the dataset identifier PXD023915.

### 2.4. Statistical Analysis

All results are presented as mean ± SD. Independent *t*-test, Kruskal–Wallis tests, or Mann–Whitney *U* tests were used for comparison studies. Statistical analysis was performed with SPSS 22. A *p*-value of <0.05 was considered statistically significant.

## 3. Results

### 3.1. Subject Characteristics

Among the 110 subjects in the dataset, a total of eight age-matched AIS subjects with a maximal Cobb angle of <30° at the first visit (baseline) without any prior treatment and who were skeletally immature (years since menarche of <two years; Risser sign of ≤four) were included in this study. The selection criteria for the AIS NP and AIS P subgroups and the clinical parameters are described in Table 2. None of the NP subjects received bracing or any treatment during the follow-up period, while all of the P group subjects received bracing treatment but still progressed and reached the surgical threshold. As shown in Table 1 and Table 2, AIS subjects in the P group had a significantly later onset of menarche, lower tanner staging, and were skeletally less mature compared to the NP group, despite having a similar chronological age. The lean mass of the right and left arms and the trunk was statistically lower in the P group compared to the NP group. On the contrary, skeletal muscle mass, body fat mass, fat-free mass, right and left leg lean mass, and handgrip strength did not show a significant difference between the two AIS subgroups. Body weight, body height, arm span, and body mass index (BMI, calculated as body weight/arm span^2^ [7]) showed no significant differences between the NP and P groups, although body weight, body height, and BMI were found to be significantly lower in the P group than in the CTRL group. The Cobb angle of the major curve in the two AIS groups showed no significant difference at their first visit.

### 3.2. Up- and Downregulated DEPs in Pairwise Comparison Analysis

**Figure 2:**
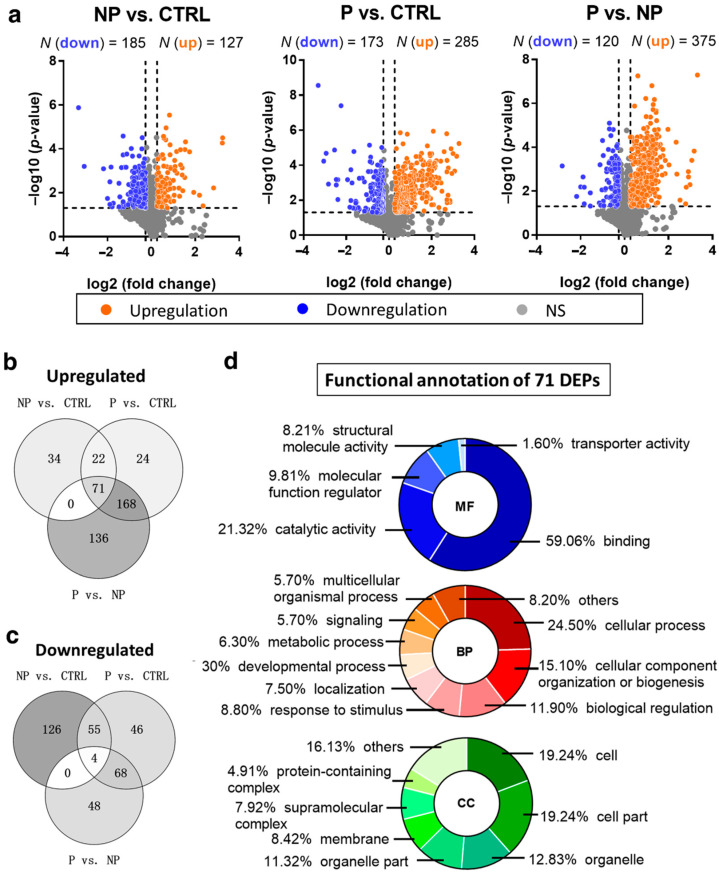
Analysis of differentially expressed proteins (DEPs). (**a**) Volcano plot of the relative quantitation results. A *t*-test was used between groups. Proteins with a ratio of ≤0.83 or ≥1.2 and a *p*-value < 0.05 were considered as DEPs. Blue and orange plots indicate down and upregulated DEPs, respectively. Gray plots represent non-significant (NS) proteins. (**b**,**c**) Venn diagrams show the overlap among all down and upregulated DEPs in the three comparison groups: NP vs. CTRL, P vs. CTRL, and P vs. NP. (**d**) PANTHER Gene Ontology (GO) functional categorization of upregulated DEPs presented in all of the comparison groups in (**b**). MF, BP, and CC represent the GO aspects molecular function, biological process, and cellular component, respectively. The percentage of each term was calculated as a protein hit the term/total function hits. The BP and CC terms less than 4% were combined as “others”.

In total, 34,915 spectra were matched to 4749 peptides, and 1058 proteins were identified in all three groups of plasma samples (Table S1). Compared to the healthy control group, the non-progressive group had 127 upregulated and 185 downregulated DEPs, while the progressive group had 285 upregulated and 173 downregulated DEPs (Figure 2a). Compared to the non-progressive group, there were 375 proteins upregulated and 120 downregulated in the progressive group.

### 3.3. Gradually Changing DEPs Accompanying a Higher Risk of Scoliosis Progression

In order to discover those circulating DEPs that had gradually higher or lower levels accompanying a higher risk of scoliosis progression, the overlap of up and downregulated DEPs in all three comparison groups was determined. Only four proteins, namely, AIM1L (absent in melanoma 1-like), SOX2 (SRY-box 2), WDR7 (WD repeat domain 7), and DNM3 (dynamin 3), showed decreasing levels when comparing NP vs. CTRL, P vs. CTRL, and P vs. NP (Figure 2b). AIM1L, known as CRYBB2 (beta/gamma crystallin domain-containing protein 2), is biased in terms of expression in the esophagus and has functions in carbohydrate binding. WDR7 is a component in synaptic vesicles and is functionally involved in hematopoietic progenitor cell differentiation. DNM3 is a microtube-associated force-producing protein involved in producing microtubule bundles. SOX2 is a critical transcription factor regulating early embryogenesis.

On the contrary, 71 proteins showed upregulation when comparing NP vs. CTRL, P vs. CTRL, and P vs. NP (Figure 2c). GO analysis with PANTHER showed the distribution of these DEPs’ annotation to be in three functional aspects (Figure 2d). For the molecular function (MF), the term “binding”—which includes protein–protein, protein–nucleic acid, and protein–lipid binding—was the most annotated term (59.06%). Catalytic activity was the second most annotated term (21.32%), which could indicate that some of the DEPs were enzymes. “Cellular process” (24.54%) was the most annotated biological process (BP) aspect, representing any process carried out at the cellular level, such as processes in a single cell, or cell–cell communication occurring at the cellular level. This term was followed by “cellular component organization or biogenesis” (15.10%) and “biological regulation” (11.90%). For the cellular component (CC), “cell” and “cell part” (both 19.24%) were the most annotated, followed by “organelle” and “organelle part” (12.83% and 11.32%, respectively).

### 3.4. Shortlist of Muscle-Related DEPs

**Figure 3:**
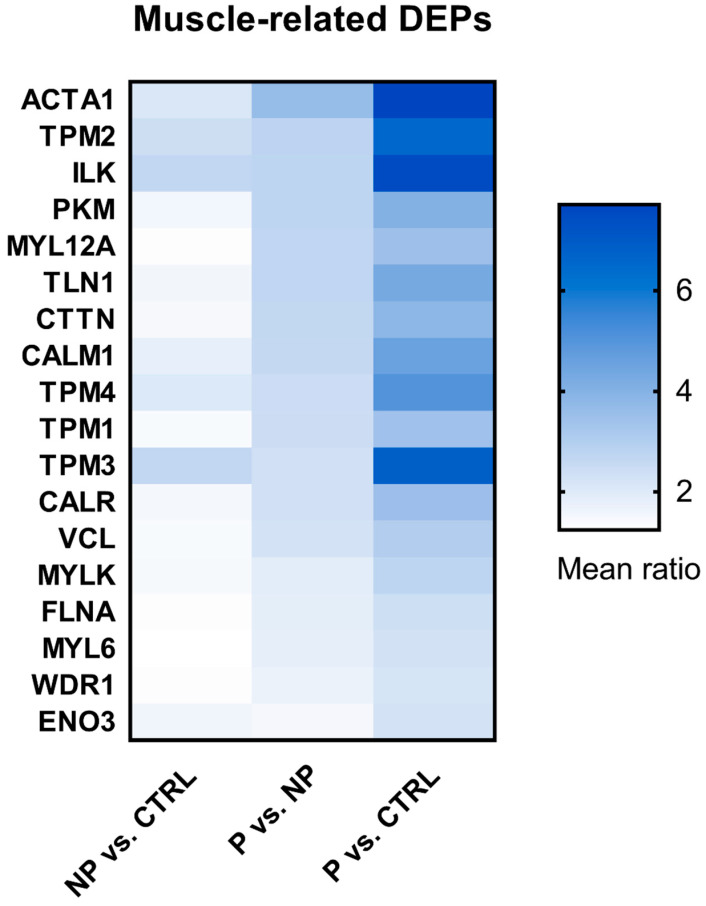
Heatmap of the upregulated DEPs that are functionally related to muscle. The DEPs are listed, sorted by the mean ratio of the P vs. NP groups. The mean ratio was calculated as the mean of four ratio values, e.g., the mean ratio of NP vs. CTRL = (NP_1/CTRL_1 + NP_1/CTRL_2 + NP_2/CTRL_1 + NP_2/CTRL_2)/4.

The gradually changed DEPs accompanying a higher risk of curve progression were analyzed using DAVID to determine their detailed function. None of the four gradually downregulated DEPs in Figure 2b were directly relevant to muscles according to GO annotation. Meanwhile, among the 71 upregulated DEPs in Figure 2c, 19 of them could annotate BP terms directly related to muscle activities in terms of including the keyword “muscle” (Table S2), such as “muscle contraction” and “muscle filament sliding”. Due to the structural and functional similarities of skeletal, cardiac, and smooth muscle, many of the proteins can be expressed in more than one type of muscle. Calmodulin 1 (CALM1), for example, is expressed in all kinds of muscle cells and could regulate their contraction. Therefore, the GO terms related to all three muscle types were retained in our analysis. One of the DEPs, named angiotensinogen, which can annotate the GO term “response to muscle activity involved in regulation of muscle adaptation”, was excluded from our list, since it was known to be generated and secreted by the liver and thus its modulating effect on muscle function is endocrine in nature only. The remaining 18 DEPs (Figure 3) were considered to be directly involved in muscle activities, while their circulating levels were associated with the risk of scoliosis progression.

### 3.5. PPI Network of 18 Candidate DEPs

**Figure 4:**
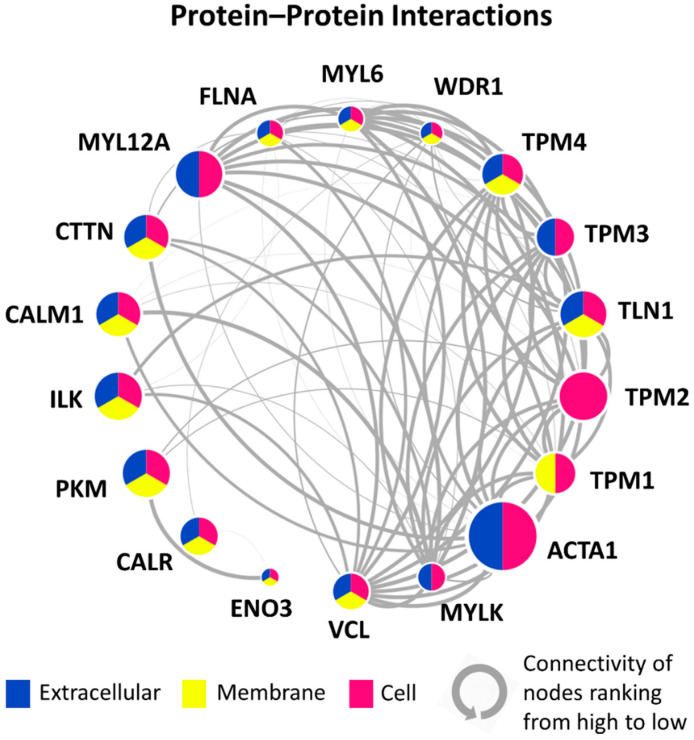
Protein–protein interaction (PPI) network among the upregulated muscle-related DEPs. The colors of each node (DEP) represent its location. The size of the node indicates its mean ratio of the P vs. NP groups. The nodes are sorted from VCL to ENO3 by the degree of connectivity calculated by Cytoscape and listed in a circle. The line (interaction) thickness indicates the interaction score calculated by STRING, which represents the strength of data support.

A PPI network of the 18 shortlisted DEPs was developed (Figure 4). The lines (interaction) thickness indicates the interaction score (range = 0.403~0.999) calculated by STRING, which represents the strength of data support. The size of the nodes (DEPs) indicates the mean ratio of the P vs. NP groups. ACTA1 (α-actin) had the largest expression ratio between the P vs. NP groups. The location of the DEPs was determined according to GO annotation and is represented by three colors. “Extracellular” represents all extracellular regions, including the extracellular matrix. “Membrane” includes both the membrane part and the cell junction. “Cell” indicates all parts inside the cell, such as the organelle part. The nodes were sorted by the degree of connectivity calculated by Cytoscape and listed in a circle. For example, VCL (vinculin), which is involved in cell matrix adhesion and cell–cell adhesion, had the highest connectivity, as it was shown to interact with 13 DEPs. The complex interactions among these DEPs indicate they are closely connected with one another and function as a network.

## 4. Discussion

As previous proteomic studies have only compared the profiles between the control and AIS groups [20,21], the current comparison analysis attempted to move a step further to compare the circulating protein profiles between healthy girls and girls with AIS, who were further divided into non-progressive and progressive AIS subgroups. This preliminary study demonstrated that the baseline muscle-related DEPs in the circulation of AIS subjects might represent a novel group of biomarkers for predicting curve progression to the surgical threshold.

A number of circulating proteins have been proposed as prognostic factors of AIS, such as melatonin and calmodulin [33,34,35,36,37]. Recently, our group proposed a composite model composed of plasma miR-145 and total procollagen type 1 N-terminal propeptide (P1NP) together with clinical parameters to predict the risk of curve progression in AIS, achieving a sensitivity of 72.2% and a specificity of 90% [38]. Despite there being a close interaction between bone and muscle, there are, as of yet, no muscle-related biomarkers for AIS. This proteomic study showed that among all of the upregulated DEPs in the progressive group, approximately 25% of DEPs (18 of 71) are found in muscles and play essential roles in muscle activities, such as muscle contraction and muscle regeneration. Among them, ACTA1 showed the highest fold change in the progressive vs. non-progressive AIS comparison, followed by another structural protein, TPM2 (tropomyosin beta chain). ACTA1 and TPM2 are only expressed in striated muscle. ACTA1 in the circulation is a well-reported candidate for muscle damage caused by physical damage or intensive exercise [39,40,41,42]. Injury could lead to an increase (up to 187 times) in ACTA1 levels in the serum [40]. Collectively, our preliminary results first suggest that there might be muscle structural damage to a certain extent in patients with AIS, which leads to the release of some cellular proteins into the bloodstream. Further in-depth investigation is warranted to determine whether these structural proteins originate from paraspinal muscles under asymmetric tension or a global phenomenon. In addition to ACTA1, some other DEPs are also associated with the etiopathogenesis of muscle-related diseases. ENO3 (beta-enolase) and its mutations can cause glycogen storage disorder XIII (GSD13, also known as Enolase-beta deficiency), which presents myalgia post exertion in adulthood and recurrent rhabdomyolysis [43]. Mutations of TPM2 and TPM3 (tropomyosin 3) have been reported to cause various congenital myopathies, such as CAP myopathy, Nemaline myopathy types 1 and 4, and congenital myopathies with fiber-type disproportion [44]. The close interactions among the 18 DEPs shown in the PPI network indicate that they function as a network; thus, the alternation of muscle fibers might not only be limited to structure, but also to affected muscle functions in patients with AIS. Most importantly, the upregulation of these DEPs in AIS in the progressive group was shown to have occurred prior to the Cobb angle progressing into a severe curvature, as the plasma were all collected at baseline, at which both the progressive and non-progressive AIS subjects had similar curve severities. Of note, the concurrent lower trunk and arm lean mass observed in the progressive group is in line with an abnormal muscle-related proteomic profile, further supporting the likelihood of impaired muscle functioning in the progressive group in the early stage.

The present study has its clinical implications. First, given the etiology of AIS remaining undetermined, the novel muscle-related DEP profiles observed in this study strongly indicate primary pathological changes in muscle tissues in AIS subjects, thus suggesting a new perspective for therapy to target the underlying etiology. Second, these proteomic markers from patients in the early stage of AIS provide potential predictors for curve progression. From the view that patients with AIS are recommended to visit the clinic regularly (e.g., every six months) until skeletal maturity to monitor curve progression, the predictors presented in this study could be helpful in risk stratification of AIS so that unnecessary clinical visits can be minimized, especially under conditions such as the COVID-19 pandemic. Third, screening muscle-related proteins in plasma samples has the potential to replace repeated X-ray measurements, which cause radiological toxicity, though further validation with larger sample sizes is warranted.

This study has some advantages compared to previous studies. (1) In order to perform a strict subgrouping of non-progressive AIS, the current study performed a six-year longitudinal follow-up until the patients reached skeletal maturity. (2) There have been studies reporting abnormal muscle morphology and functions in AIS, especially at paraspinal regions of the major curve. Nevertheless, these cross-sectional studies failed to answer whether the abnormal muscle phenotypes are primary or secondary to spinal curvature. Our study, conducted at baseline when the AIS subgroups had comparable severity, provides fresh evidence indicating the likelihood of abnormal muscle phenotypes at the protein level in AIS, which might contribute to curve progression. (3) iTRAQ-based proteomic analysis enables the simultaneous screening of thousands of proteins reflecting systemic changes of the whole body, and thus could provide enough candidates for further evaluation of biomarkers and for the establishment of a predication model of curve progression.

However, there are limitations in this study. (1) The findings of this pilot study, with a limited sample size in non-progressive and progressive AIS groups, requires further validation with larger and longitudinal cohorts. (2) Only female subjects were recruited in this study, which could have caused sampling bias. Despite there being higher AIS prevalence and risk of curve progression in girls, further investigations with cohorts including boys are necessary to validate our findings and to identify sex-specific DEPs in AIS. (3) Progressive and non-progressive subjects of similar ages and Cobb angles at the baseline were selected to minimize individual variation. However, differences in sexual and skeletal maturity between these two groups still exist and might be a cofounding factor partly contributing to the profile of DEPs. It should be noted that these unmatched factors might also contribute to the risk of progression, which should be taken into consideration in future similar studies. (4) Despite technological advancement, iTRAQ is still a costly approach when compared to well-established but less-sensitive methods such as 2D difference gel electrophoresis [45]. Therefore, the strategy of pooling plasma samples within one group was adopted, which is a common approach to reducing the variance of biological replicates in proteomics studies due to small sample sizes [46]. However, the pooling strategy has its own shortages, such as the effect of outlier samples. Therefore, verifying the levels of candidates in individual samples is desired in future validation studies.

In summary, this is the first study to hypothesize that there might be a higher level of circulating baseline muscle-related proteins in AIS, which might further link to the risk of curve progression. Herein, we proposed 18 proteins as potential candidates reflecting alternations of muscle phenotypes, which require further investigation to verify the circulating levels and biological functions of these biomarker candidates, and eventually a refined composite model for curve prediction.

## Figures and Tables

**Table 1:** Basic information for the three groups.

Groups	CTRL (*N* = 50)	NP (*N* = 4)	P (*N* = 4)	*p*-Value ^1^
Age (years)	13.1 ± 0.5	13.4 ± 0.5	12.6 ± 0.9	0.155
Body Weight (kg)	49.6 ± 9.8	42.2 ± 3.2	34.3 ± 3.8 *	0.002
Body Height (cm)	157.4 ± 6.1	154.0 ± 10.6	145.8 ± 3.4 *	0.014
Arm Span (cm)	156.1 ± 6.9	152.4 ± 12.2	146.9 ± 8.1	0.090
BMI (kg/m^2^)	20.23 ± 3.0	18.3 ± 2.3	15.9 ± 0.5 *	0.005

Abbreviations: CTRL, healthy control; NP, non-progressive AIS; P, progressive AIS. Data are shown as mean ± SD. ^1^ Kruskal–Wallis test was used. * *p* < 0.05 when compared to the CTRL group using a post hoc test.

**Table 2:** Selection criteria for the AIS subgroups and the subjects’ information.

AIS Subgroups	Non-Progressive (NP)		Progressive (P)		
	Mean	SD	Mean	SD	*p* ^1^
Selection criteria of the subgroups					
Cobb at first visit (°)	23.5	2.4	26.5	3	0.189
	(Individual: 21, 22, 25, 26)		(Individual: 23, 25, 29, 29)		
Change of Cobb angle at latest follow-up	Less than 6°		Final Cobb larger than 45°		/
	(Individual: 24, 21, 20, 28)		(Individual: 57, 59, 49, 50)		
With bracing	No (0/4)		Yes (4/4)		/
Sexual and skeletal maturity					
Time since onset of menarche (years)	1.6	0.8	−0.9	0.4	0.021
	(range: 0.62~2.57)		(range: −1.38~−0.34)		
Breast stage	3.3	0.5	2.0	0.8	0.044
	(range: 3~4)		(range: 1~3)		
Pubic hair	3.0	0.0	1.8	0.5	0.011
	(range: 3~3)		(range: 1~2)		
Risser sign	3.5	0.6	0.8	1.0	0.019
	(range: 3~4)		(range: 0~2)		
Body composition					
Skeletal muscle mass (kg)	16.8	2.7	14.1	1.2	0.149
Body fat mass (kg)	9.9	2.1	7.1	2.0	0.149
Fat-free mass (kg)	32.0	4.7	27.5	2.2	0.149
Right arm lean mass (kg)	1.2	0.2	0.9	0.1	0.029
Left arm lean mass (kg)	1.2	0.2	0.9	0.1	0.043
Trunk lean mass (kg)	13.4	1.4	10.9	0.8	0.043
Right leg lean mass (kg)	4.9	1.2	3.8	0.3	0.083
Left leg lean mass (kg)	4.8	1.1	3.7	0.3	0.083
Handgrip strength					
Dominant hand (kg)	17.8	3.9	14.4	3.4	0.146
Non-dominant hand (kg)	18.4	5.3	13.8	3.6	0.149

^1^ Mann–Whitney *U*-test was used. *p* in bold indicated a value of <0.05.

## Data Availability

The mass spectrometry proteomics data were deposited to the ProteomeXchange Consortium via the PRIDE partner repository with the dataset identifier PXD023915.

## Supplementary Materials

The following are available online at https://www.mdpi.com/article/10.3390/jcm10214927/s1. Table S1, All identified proteins with quantitative data; Table S2, Muscle activity-related GO terms in the aspect of biological function annotated by 19 upregulated DEPs.
